# Segregation between *SMCHD1* mutation, D4Z4 hypomethylation and Facio-Scapulo-Humeral Dystrophy: a case report

**DOI:** 10.1186/s12881-016-0328-9

**Published:** 2016-09-15

**Authors:** Marie-Cécile Gaillard, Francesca Puppo, Stéphane Roche, Camille Dion, Emmanuelle Salort Campana, Virginie Mariot, Charlene Chaix, Catherine Vovan, Killian Mazaleyrat, Armand Tasmadjian, Rafaelle Bernard, Julie Dumonceaux, Shahram Attarian, Nicolas Lévy, Karine Nguyen, Frédérique Magdinier, Marc Bartoli

**Affiliations:** 1 Aix Marseille Univ, INSERM, GMGF, Marseille, France; 2 APHM, Centre de Référence des Maladies Neuromusculaires et de la SLA, Hôpital de la Timone, Marseille, 13385 France; 3 Center of Research in Myology/ Institut de Myologie UMR974 - UPMC Université Paris 6/ Inserm /FRE3617– CNRS, Groupement Hospitalier de la Pitié Salpétrière, Paris, Cedex 13 France; 4 APHM, Laboratoire de Génétique Médicale, Hôpital de la Timone, Marseille, 13385 France

**Keywords:** Facio-Scapulo-Humeral Dystrophy, DNA methylation, SMCHD1, DNA combing, Haploinsufficiency, DUX4

## Abstract

**Background:**

The main form of Facio-Scapulo-Humeral muscular Dystrophy is linked to copy number reduction of the 4q D4Z4 macrosatellite (FSHD1). In 5 % of cases, FSHD phenotype appears in the absence of D4Z4 reduction (FSHD2). In 70-80 % of these patients, variants of the *SMCHD1* gene segregate with 4qA haplotypes and D4Z4 hypomethylation.

**Case presentation:**

We report a family presenting with neuromuscular symptoms reminiscent of FSHD but without D4Z4 copy reduction. We characterized the 4q35 region using molecular combing, searched for mutation in the SMCHD1 gene and determined D4Z4 methylation level by sodium bisulfite sequencing. We further investigated the impact of the *SMCHD1* mutation at the protein level and on the NMD-dependent degradation of transcript.

In muscle, we observe moderate but significant reduction in D4Z4 methylation, not correlated with *DUX4-fl* expression. Exome sequencing revealed a heterozygous insertion of 7 bp in exon 37 of the *SMCHD1* gene producing a loss of frame with premature stop codon 4 amino acids after the insertion (c.4614-4615insTATAATA). Both wild-type and mutated transcripts are detected.

**Conclusion:**

The truncated protein is absent and the full-length protein level is similar in patients and controls indicating that in this family, FSHD is not associated with SMCHD1 haploinsufficiency.

**Electronic supplementary material:**

The online version of this article (doi:10.1186/s12881-016-0328-9) contains supplementary material, which is available to authorized users.

## Background

FSHD is one of the most common hereditary neuromuscular disorders, affecting between 1 in 8300 to 1 in 20,000 people in different Western populations [1, 2]. The disease is marked by clinical variability in disease onset and penetrance. The clinical phenotype is characterized by the progressive involvement of specific facial, scapulohumeral and anterior foreleg muscles. Muscle weakening is frequently asymmetric and can spread to the pelvic girdle, abdominal and anterior lower leg muscles in most severe cases [3]. At the genetic level, this disease is transmitted as an autosomal trait. In around 95 % of patients, FSHD is associated with a reduction in the number of copies of a 3.3 kb tandem macrosatellite element, D4Z4 on one of the two 4q35 alleles. In the unaffected population, D4Z4 arrays comprises 11 to 150 units while the number of copies ranges between 1 to 10 in FSHD patients [4]. In most cases, the D4Z4 contraction is pathogenic if it segregates in *cis* with a specific distal polymorphic sequence on 4q35, termed 4qA, which is present in ~50 % of the chromosomes 4 in the human population [4]. In 5-10 % of families with a typical FSHD phenotype, there is no linkage to D4Z4 shortening and this type of FSHD is referred to as type 2 (FSHD2; OMIM 158901). Approximately 80 % of FSHD2 individuals carry a mutation in the epigenetic modifier, Structural Maintenance of Chromosomes Flexible Hinge Domain Containing 1 gene (*SMCHD1*) on chromosome 18 [5] often associated with hypomethylation of the D4Z4 element, mainly in the proximal end of the D4Z4 repeat (*Fse*I restriction site <25 % [5]; DR1 sequence [6], <30 %; 5P proximal sequence < 55 % [7]). Several groups have identified variants in the *SMCHD1* gene that include alteration of splice sites, insertions, deletions, or missense and nonsenses [8–13].

The *SMCHD1* gene encodes a 226KDa protein containing a GHKL-type ATPase domain and a hinge domain. The SMCHD1 protein belongs to the “Structural Maintenance of Chromosomes” family, which includes seven members (SMC1A, 1B-6). In mice, this protein binds to the PRC2 polycomb complex and colocalizes with trimethylH3K27 or associates with Dnmt3B for X chromosome inactivation and variegation of gene expression [14–16]. In FSHD, SMCHD1 loss of function, dominant negative effect or haploinsufficiency might be associated with D4Z4 hypomethylation, chromatin relaxation and ectopic expression of the long form of the *DUX4* transcript (DUX4-fl) encoded by the last D4Z4 repeat and the flanking 4qA region [17].

We identified a family in which the proband carries a 7-nucleotide insertion in exon 37 of the *SMCHD1* gene. We determined the segregation of the mutation in the family, its functional consequence at the mRNA and protein levels in peripheral blood mononuclear cells (PBMCs), fibroblasts and muscle together with association with D4Z4 methylation and *DUX4* expression.

## Methods

### Sample collection

Individuals were clinically assessed by neurologists with expertise in neuromuscular diseases who defined the presence or total absence of clinical signs and evaluated the involvement of the typical groups of muscle usually affected in the disease (facial, shoulder and pelvic girdle, upper and lower limbs and abdominal muscles). Based on the recent CCEF classification of FSHD patients, the proband was classified in category A2 (upper and lower facial weakness, upper limb impairment and winged scapula) [18]. Research was approved by a local ethic committee. Patients and relatives have provided written informed consent for the use of the blood samples, tissues and DNA for medical research. Written informed consent was obtained from the patient for publication of this Case report and any accompanying images. A copy of the written consent is available for review by the Editor of this journal. Research was performed in accordance with the Declaration of Helsinki.

### Cell culture

Skin biopsy was obtained using standard procedures. Primary fibroblasts were obtained by placing the skin biopsy in a culture dish containing DMEM medium supplemented with 4.5 g/L of glucose, 2 mM glutamin, 10 % fetal calf serum (FCS) and 1 % Penicillin/Streptomycin for 15 days. At subconfluence, primary cells were collected by addition of 0.25 % trypsin and 1 mM EDTA, resuspended in fresh DMEM medium, plated and incubated at 37 °C in 5 % CO_2_.

### DNA, RNA and protein extraction

Total DNA was extracted from peripheral blood mononuclear cells (PBMCs) using the Qiagen DNeasy Blood & Tissue Kit, following manufacturer’s instructions. Total RNA was extracted from peripheral blood with Trizol-Chloroform (Life Technologies) following manufacturer’s instructions. After DNAse treatment (Ambion), total RNA was converted to cDNA using the High-Capacity cDNA Reverse Transcription Kit (Applied Biosystems) with random primers (Life Technologies). Whole protein extracts were obtained from cells disrupted in 200 μL extraction buffer (Tris-HCl pH8 100 mM, 10 % SDS, 10 mM EDTA, 10 % glycerol, protease inhibitors).

### Western blotting

Proteins were separated by electrophoresis and transferred onto a PVDF membrane following the protocol recommended by the supplier for the Life Technologies NuPAGE system for Bis/Tris 4-12 % gels. After transfer, PVDF membranes were blocked for 1 h in 5 % (w/v) non-fat dry milk in PBS-T (0.1 % Tween-20 in PBS) and incubated for 90 min with either Lamin B (Abcam, EPR9701), N-terminal SMCHD1 (Sigma, HPA039441) or C-terminal SMCHD1 (Abcam, ab31865). After 4 washes in PBS-T, membranes were incubated for 90 min with anti-mouse IgG secondary antibody coupled to HRP (1/20 000; ThermoFisher). Signals were revealed using enhanced chemiluminescence (Immobilion Western, Milipore) using a ChemiDoc XRS system (Bio-Rad).

### Molecular combing

Experiments were performed as described by Nguyen et al, [19] on peripheral blood mononuclear cells (PBMCs) embedded in agarose plugs. After purification, DNA was diluted in MES buffer and combed on coverslips. A set of probes specific for either the genomic organization of the 4q or 10q subtelomeric regions is used allowing bar coding of the two regions and measurement of the D4Z4 array (Additional file 1: Figure S1). After hybridization, the entire coverslip is scanned by an automated fluorescence microscope and image analysis is performed using the Combilog software. Only intact D4Z4 signals are kept for analysis.

### Exome sequencing and PCR

Exome capture with Agilent SureSelect All Exon kit V5 followed by paired-end sequencing with HiSeq2000 was applied to DNA samples from patients II.1 and I.1. Sequencing was performed by IntegraGen SA (Evry, France). Other muscular gene variants were selected from a restricted list of 45 genes currently used for neuromuscular disease diagnosis. All variants, listed in Additional file 2: Table S1, had a genetic status in proband and mother consistent with disease age of appearance and/or severity, and a frequency lower than 5 % when present in dbSNP database. *In silico* predictions were performed with HSF3.0 [20] and UMD predictor [21]. Finally, presence of variants was assessed in HGMD, LOVD and ClinVar (Additional file 2: Table S2).

All primers were designed using the Primer3 software (http://frodo.wi.mit.edu/) and checked by BLAST against the human genome to ensure specificity and SNPcheck (https://secure.ngrl.org.uk/SNPCheck/snpcheck.htm;jsessionid=8E9E8C73969EB4D2) to avoid allele drop-out. Primers used for *SMCHD1* exon 37 amplification and Sanger sequencing are the following: forward 5’- TGC-CTG-TGG-AAC-ACT-CAA-AC-3’ and reverse 5’-GCT-GAC-TTC-CCA-ATT-TAG-TGC-3’. Reactions (95 °C for 20 s, 59 °C for 40 s and 72 °C for 1 min and 40 s) were run for 35 cycles. PCR products were purified by Exonuclease I (New England Biolabs) and Rapid Alkaline Phosphatase (Roche Biochemicals) digestion, subjected to Big Dye Terminator v3.1 sequencing reaction (Applied Biosystems) and analyzed by 3130xl Genetic Analyzer (Applied Biosystems) following manufacturer’s instructions.

### Sodium bisulfite sequencing

For bisulfite modification, 2 μg of genomic DNA was denaturated for 30 min at 37 °C in NaOH 0.4 N and incubated overnight in a solution of sodium bisulfite 3 M pH5 and hydroquinone 10 mM using a previously described protocol [22]. Converted DNA was amplified using primer sets already reported [6, 7]. Amplification was carried out using the High Fidelity Taq polymerase (Roche) following manufacturers’ instructions. PCR products were purified using the Wizard SV gel and PCR Purification system (Promega) and cloned using the pGEM**®**-T Easy Vector cloning kit (Promega). After overnight incubation at 37 °C with antibiotic selection, at least ten randomly selected clones were PCR amplified for each sample using T7 and Sp6 primers and sequenced using by Sanger’s method (Eurofins MWG Operon, Ebersberg, Germany) with either Sp6 or T7 primers. Sequences were analyzed using the BiQ Analyzer software [23] and the average methylation score was calculated as the number of methylated CpGs for the total number of CpGs in the reference sequence.

### RT-PCR and data analysis

The *XNP* transcript was amplified for all cDNA samples in order to check for genomic contamination using the following primers: forward 5’- AGG-AAA-GGC-AGG-TGC-AAA-GC-3’ and reverse 5’- CGG-AGC-TTA-AAC-TCA-TGG-AGG-3’. *SMCHD1* transcript was amplified for all cDNA samples with forward 5’- AAT-GTT-CGC-TCA-GTT-GCC-AG-3’ and reverse 5’- AGG-ACT-ACT-TTC-TGC-CAG-CA-3’ primers for 35 cycles (95 °C for 30 s, 58 °C for 1 min and 72 °C for 2 min). PCR products from sample II.1 and controls were subcloned in the pGEM**®**-T Easy Vector cloning kit following manufacturer’s instructions (Promega) and Sanger sequenced with Sp6 and T7 forward specific primers. DUX4 *expression* was analyzed as described [24].

### NMDI14 treatment and qPCR analysis

Skin fibroblasts from proband and healthy controls were grown at 37 °C and 5%CO_2_ in DMEM medium supplemented with 15 % fetal bovine serum and 1 % antibiotics (Thermo Fisher). NMDi14 (Merck Millipore) was dissolved and diluted in dimethylsulfoxide (DMSO) to a 48 mM final concentration. NMDI14 treatment on fibroblasts was performed at a 50 μM final concentration for 2 h and RNA was extracted by Trizol-Chloroform (Invitrogen) following manufacturer’s instructions. *ATF3* positive control as well as NMDI14 treatment conditions were chosen based on previous report [25]. *SMCHD1* expression was quantified by RT-QPCR using the 480 Light Cycler real-time quantitative PCR (Roche) and SYBR Green mix (Light Cycler 480 Master Mix, Roche). Primer sequences were picked in order to amplify simultaneously WT and mutated allele transcripts, while sequence similarity did not allow performing allele specific amplification. Differences in gene expression levels were determined as previously described [26].

## Case presentation

### Clinical description and molecular diagnosis

**Fig. 1:**
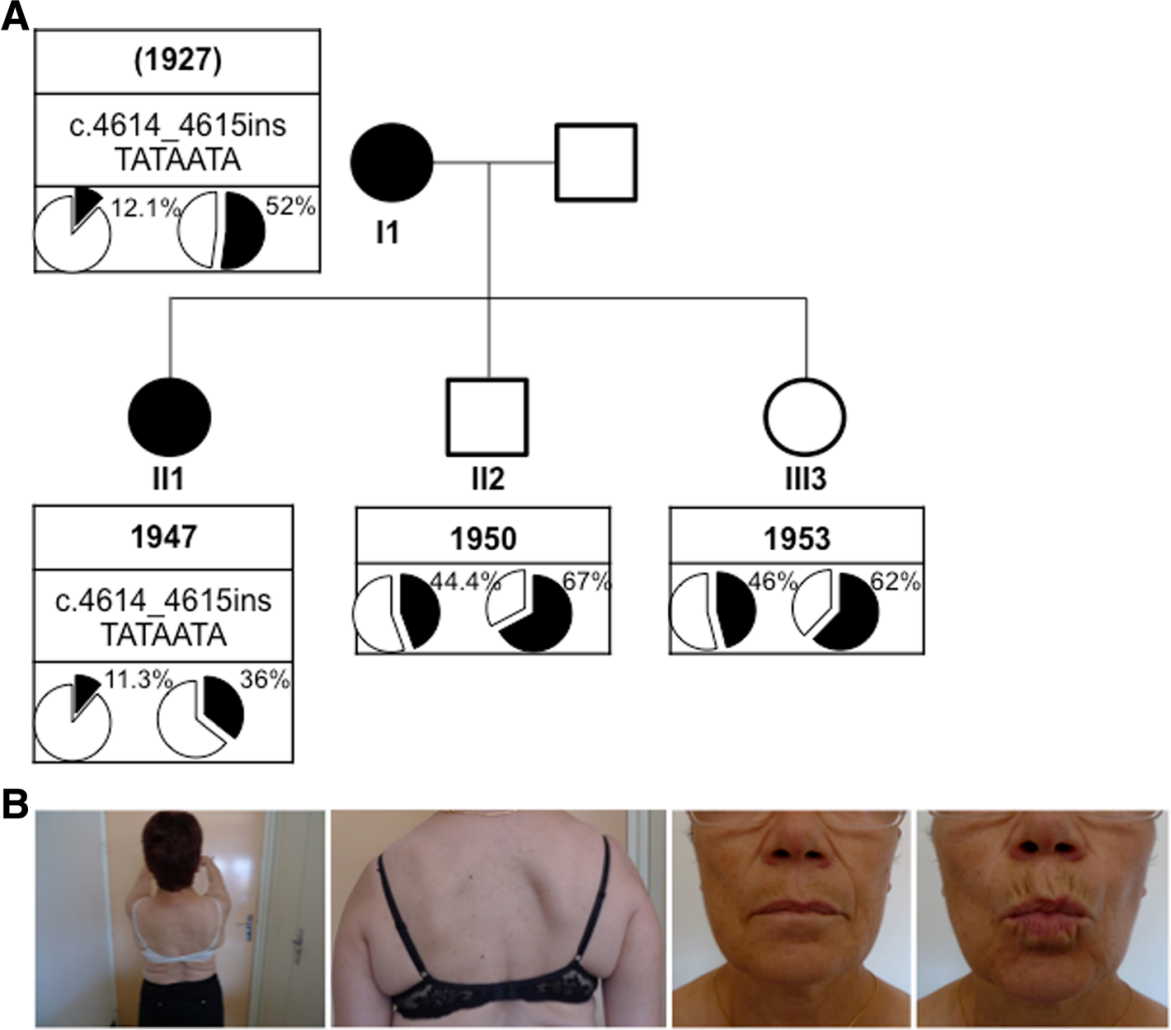
Clinical diagnosis and pedigree. **a** Pedigree of the family. For each individual year of birth is indicated together with the presence of SNP or mutation of the *SMCHD1* gene and D4Z4 methylation level (%) at D4Z4 the DR1 (left) and 5’ (right) proximal regions. Individuals I1 and II1 carry the c.4614_4615insTATAATA heterozygous *SMCHD1* mutation and display a low methylation levels compared to II2 and II3. **b** Presentation of a typical FSHD phenotype in the proband (II1) with characteristic asymmetrical scapulo humeral weakness and facial involvement

**Fig. 2:**
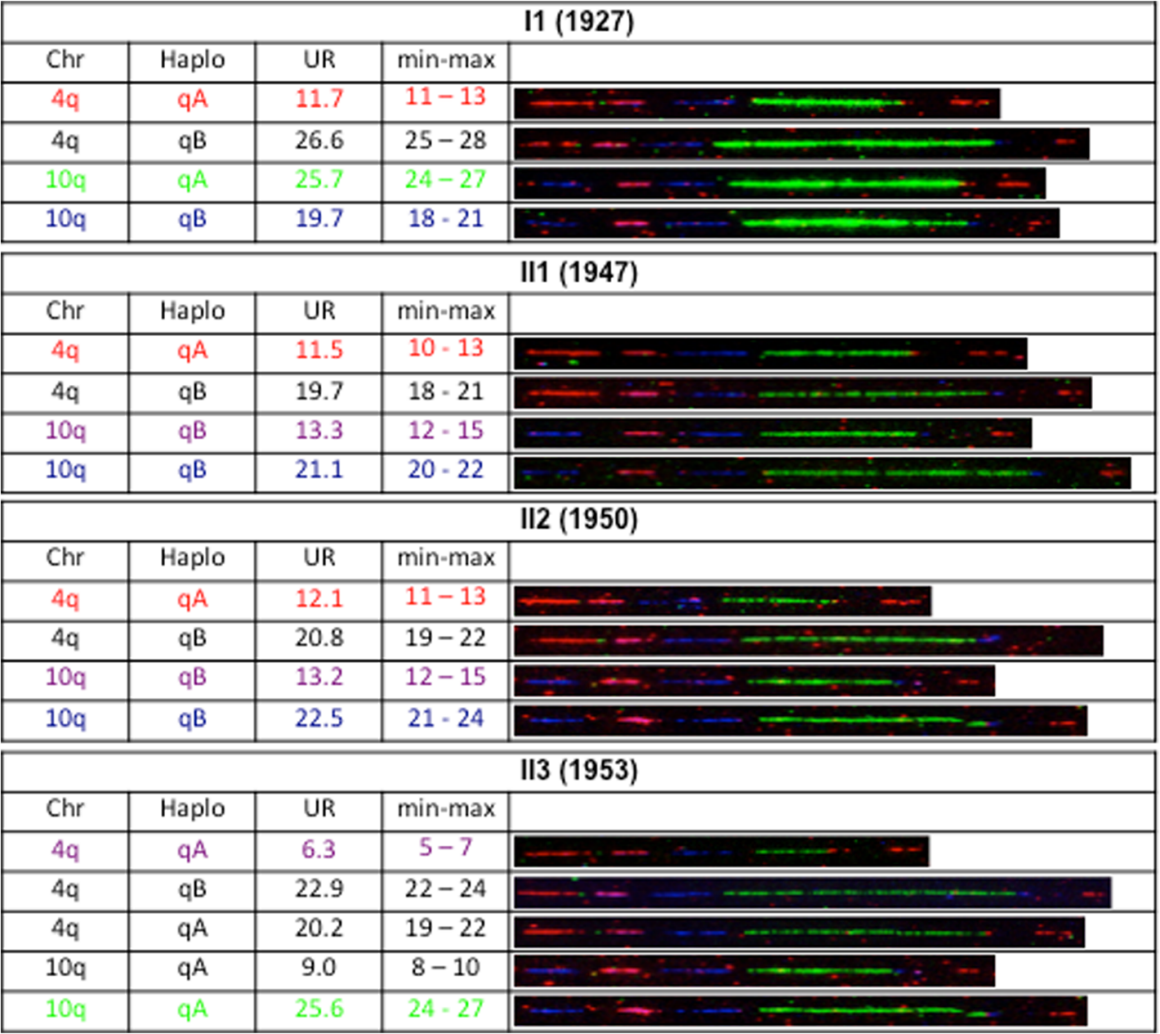
Molecular diagnosis by DNA Combing. Combed DNA from the different family members using specific probes and bar code for the 4q and 10q regions. The chromosome, haplotype and D4Z4 array size estimation (in kb) is indicated for each allele. The bar-code used to distinguish the three different alleles is based on a combination of three different colors and different DNA probes encompassing the distal regions up to the telomeric sequence [19]. The 3-color barcode comprises 2 probes detected in blue for the proximal region common to chromosomes 4 and 10, one 6 kb probe (red), which hybridizes the telomere, and a red probe that hybridizes the qA-specific β-satellite region, with a variable length (1–5 kb). The qB-specific probe, immediately adjacent to D4Z4, is detected in blue (Additional file 1: Figure S1)

The proband (II1) is a 68-years old woman who presented first signs of FSHD at the age of 56 (Fig. 1a) with lower limb weakness, stepping and frequent falls. FSHD was confirmed at the age of 61. First clinical examination confirmed facial involvement with asymmetrical smile and asymmetrical weakness of *orbiculis occuli*. In addition, the proband displayed shoulder girdle involvement and *scapula alata* (Fig. 1b), abdominal, pelvic girdle, hyperlordosis, lower limb muscles weakness, foot dorsiflexion defect and stepping. At the molecular level, analysis was first done first by Southern blotting (data not shown) and further confirmed by Molecular Combing on peripheral blood DNA (Additional file 1: Figure S1 and Additional file 3: Figure S2) [19]. She carries 12 repeated units (RU) associated to a qA haplotype on one 4q chromosome and 20 repeated D4Z4 units associated to a qB haplotype on the other allele and 13 and 21 repeated units, both associated to a qB haplotype on the 10q chromosomes (Fig. 2).

The 12 RU-4qA allele has been transmitted by her mother (I1), also affected. In this second patient, first signs appeared at the age of 45. She showed stepping at the age of 56 and became wheelchair-dependent at the age of 74. She was diagnosed at the age of 81 and displayed asymmetrical facial and upper limb weakening. The 12 RU-4qA allele has been transmitted to her son (II2; age 65) who is not affected. Interestingly, her unaffected daughter (II3; age 62) carries a complex pattern with 3 different 4q35 alleles with one qA allele with 6 RU and two other alleles with more than 11 D4Z4 units (23 RU-qB, likely inherited from the father; 20 RU-qA) suggesting respectively post and pre-zygotic mosaicism (Fig. 2). Despite the presence of a short 6 RU allele she did not show any sign of FSHD at the time of collection.

### DNA methylation analysis by sodium bisulfite sequencing and *DUX4* expression

**Fig. 3:**
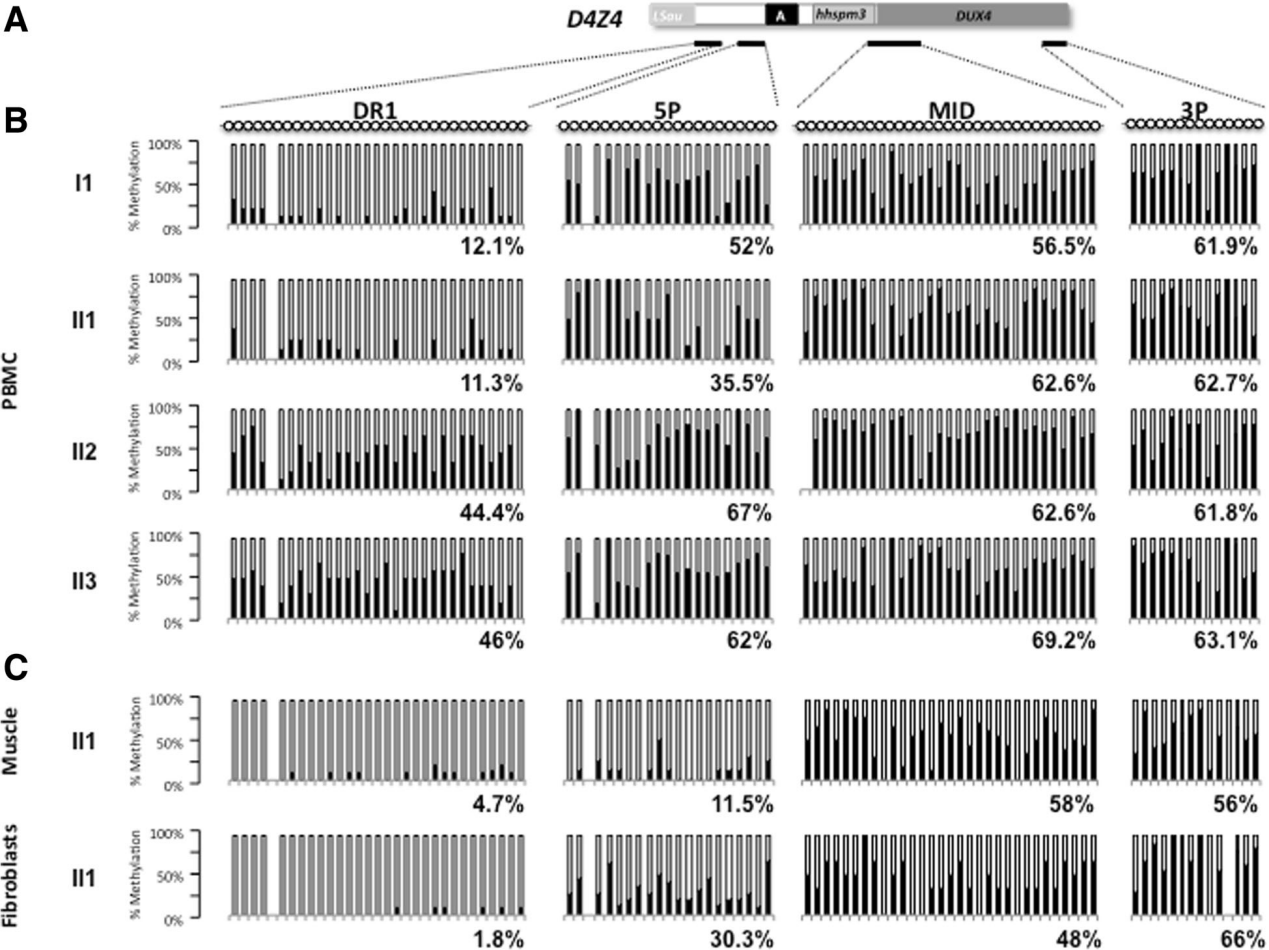
DNA methylation analysis in peripheral blood and tissues. **a** Four regions within D4Z4 were amplified by PCR after sodium bisulfite treatment of genomic DNA. Amplicons were cloned and at least 10 individual clones were analyzed by Sanger sequencing. Each clone is representative of a molecule of DNA of the initial sample. The position of the four sets or primers used is indicated with black lines below schematic D4Z4. **b** Histogram bars represent the percentage of methylated (black) or unmethylated (white) CpG for each position in the DR1 (31 CpGs), 5’ (21 CpGs), Mid (31 CpGs), and 3’ (14 CpGs) regions in genomic DNA from PBMCs for each individual. **c** DNA methylation analysis in genomic DNA from a quadriceps muscle biopsy and primary fibroblasts from the II1 index case

The level of D4Z4 methylation was analyzed by sodium bisulfite sequencing on peripheral blood DNA for the index case (II1) and the different members of the family at four different positions along the D4Z4 repeat (Fig. 3a) following same method and thresholds (below 35 % of methylated CpGs for the DR1 sequence and 55 % for the 5’ region) as reported by us and others, respectively (Fig. 3b) [6, 7].

In affected members (I1 and II1), hypomethylation in the DR1 (12.1 % and 11.3 % respectively) and 5’ (52 % and 35.5 % respectively) sequences segregates with the symptoms whereas unaffected member display higher methylation level (DR1: 44.4 % and 46 %; 5’, 67 % and 62 % for II2 and II3, respectively) (Figs. 1a and 3b). In all cases (affected or unaffected), we did not observe any significant hypomethylation for the Mid and 3’ regions (Fig. 3b). For the Mid region, the methylation level was 62.6 % (II2) and 69.2 % (II3) for unaffected members and 62.6 % (II1) and 56.5 % (I1) for affected members. For the 3’ region, the methylation level was 61.8 % (II2) and 63.1 % (II3) for unaffected members and 62.7 % (II1) and 61.9 % (I1) for affected members.

In addition, we analyzed the D4Z4 methylation pattern in DNA from primary fibroblasts and quadriceps muscle biopsy for the index case and observed that hypomethylation is more pronounced for the DR1 and 5’ regions in these two tissues compared to blood (DR1: 11.3 %, 4.7 % and 1.8 %; 5’, 35.5 %, 11.5 %, 30.3 % in blood, muscle and fibroblasts, respectively) (Fig. 3c). These data fits with previous reports by us and others with a percentage of methylated CpG below 35 % for the DR1 sequence and 55 % for 5’ [6, 7].

### Exome sequencing and pedigree segregation

Exome sequencing was performed for I1 and II1. We identified a heterozygous insertion of 7 nucleotides in exon 37 of the *SMCHD1* gene (c.4614_4615 insTATAATA) (Additional file 3: Figure S2). In order to confirm this variation and analyze segregation in unaffected members of the family (II2 and II3), we performed direct Sanger sequencing of exon 37 in all the pedigree and we determined that c.4614_4615 insTATAATA co-segregates with clinical signs of FSHD (Additional file 3: Figure S2).

In order to search for other muscular gene variants, which may contribute to the neuromuscular or FSHD phenotype, we selected variants among a list of 45 genes screened in the diagnosis of neuromuscular disorders (Additional file 2: Table S1). Interestingly, we found a novel missense heterozygous mutation in the *TTN* gene (c.8168 A > C; p.D2723A). By *in silico* analysis [20, 21], the mutation is predicted as pathogenic with a potential skipping of exon 35 and a Asp2723Ala substitution. Other gene variants, their relative genetic status and *in silico* predictions are detailed in Additional file 2: Tables S1 and S2.

### Transcripts and protein analysis

**Fig. 4:**
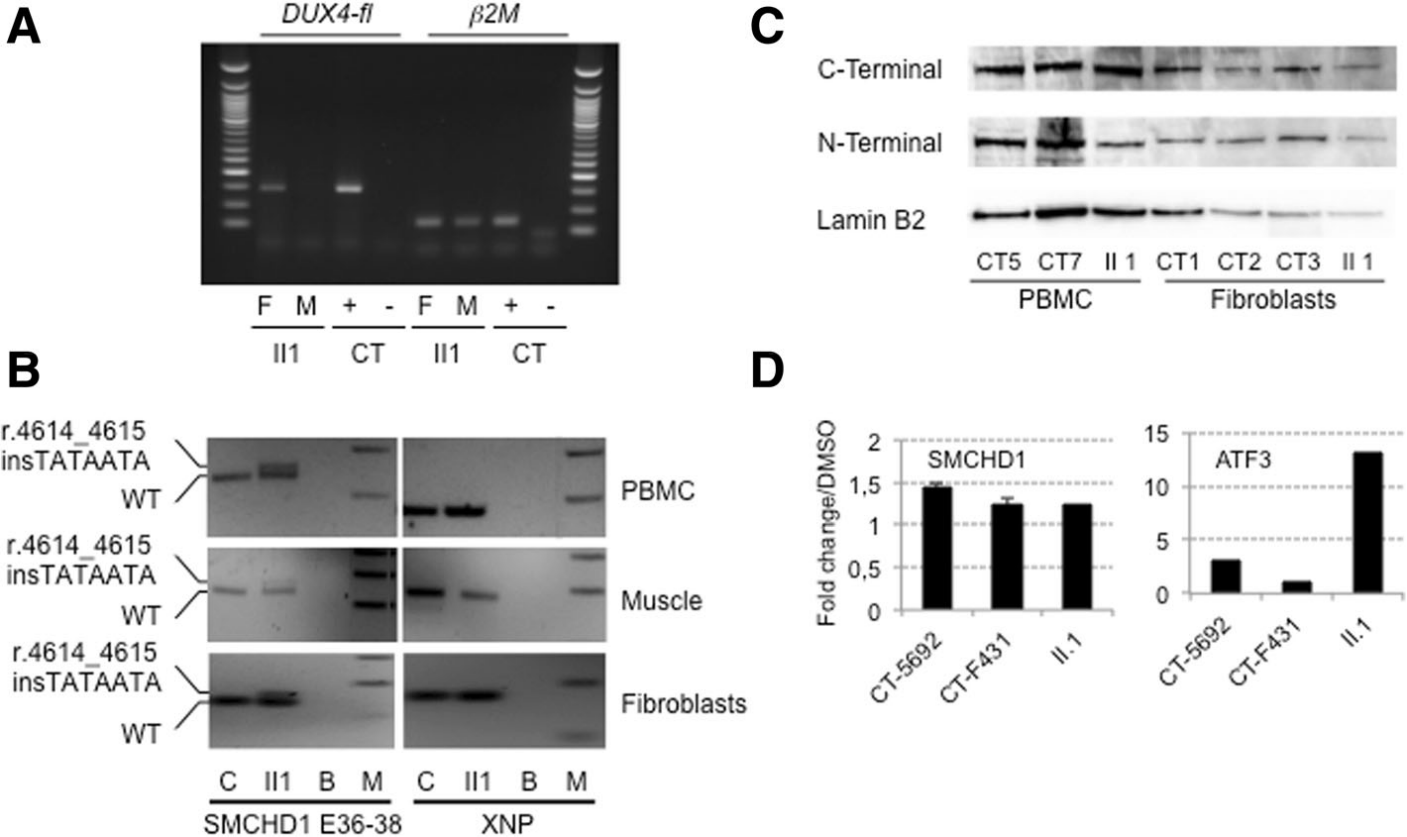
*DUX4* expression and characterization of the *SMCHD1* mutation by RT-PCR and western blotting. **a** Expression of the *DUX4* gene in total RNA from muscle (M) and primary fibroblasts (F) of the proband (II1). A positive control expressing *DUX4-fl* was used (+) and amplification was performed without reverse transcriptase (**-**). The ß2microglobulin gene was used as a standard of amplification. **b** Analysis of the *SMCHD1* transcript on cDNA obtained from II1 PBMCs, muscle biopsy and primary fibroblasts. The wild-type and transcript carrying the (r.4614_4615 insTATAATA) insertion have been amplified using primers encompassing exons 36-38. PBMCs, muscle or primary fibroblasts from healthy individual (CT) were used as controls. The XNP gene was used as a positive control. **c** SMCHD1 western blot on whole cell extracts from PBMCs and fibroblasts from the index case (II1) compared to control cells from healthy donors (CT5 and CT7 PBMC; CT1, CT2 and CT3 primary fibroblasts) with antibodies against either the N- or C-terminal epitope. The lamin B2 protein was used as loading reference. **d** Primary fibroblasts were treated for 2 h with a final concentration of 50 μM NMDi14 or mock treated with DMSO. SMCHD1 transcripts were amplified by RT-QPCR in the different conditions. The *ATF3* gene was used as a positive control [25]. Samples were amplified in triplicates

By RT-PCR (Fig. 4a) we were able to detect the DUX4-fl pathogenic transcript in cultured primary fibroblasts from the proband while the transcript was undetectable in the muscle biopsy. *SMCHD1* RT-PCR of exons 36 to 38, in II1, revealed co-expression of the wild-type allele and of the allele carrying the insertion (r.4614_4615 insTATAATA) indicating the absence of splicing defect (Fig. 4b). Moreover, results show moderate reduction in the expression of the mutated allele compared to wild-type in the 3 tissues available; PBMCs, muscle fibers and fibroblasts (Fig. 4b).

By Western blotting, we also demonstrate that the expression of *SMCHD1* is not restricted to muscle but also detectable in fibroblasts and PBMCs of control cells (Fig. 4c). However, while we looked for presence of a truncated protein (p.A1539Yfs*4) with a predicted size of 1543 residues and an expected weight of 173.57 kDa, we could not detect it by western blotting with an antibody directed against the N-terminal part of the protein either in fibroblasts or PBMC from the II1 index case. In order to determine whether the SMCHD1 mutated allele was degraded by non-sense mediated mRNA decay (NMD), we treated fibroblasts from the index case and controls for 2 h with 50 μM NMDi14 inhibitor, following previous publication [25]. Expression of the *ATF3* gene was used as a positive control of NMD inhibition [25]. Using different primers, we observed rescue of expression for SMCHD1 and ATF3 transcripts after inhibition of NMD activity in fibroblasts (Fig. 4d). However, we did not observe any significant difference in the increased level of SMCHD1 transcripts between proband and healthy control cells suggesting that the transcript carrying the 7 nt insertion is not preferentially degraded by NMD.

## Discussion

We describe here the identification of a new mutation corresponding to the insertion of 7 nucleotides in the exon 37 of the *SMCHD1* gene (c.4614_4615 insTATAATA) in a family with FSHD. The proband (II1) and her mother (I1), both showing clinical signs of FSHD, carry this SMCHD1 mutation, together with an 12 RU D4Z4 array associated with a qA haplotype. *SMCHD1* insertion is not present in the other unaffected members of the family (II2 and II3), whereas the 12 RU D4Z4 qA allele is carried by the brother (II2). Interestingly, the unaffected daughter (II3) carries three different 4q alleles, two with more than 11 repeats and one with 6 RU and a qA haplotype suggesting a complex 4q mosaicism. Nevertheless, she did not show any sign of FSHD at the time of collection.

We analyzed CpG by CpG the D4Z4 methylation level at four different positions using previously described primers (DR1; 5’, Mid and 3’) [6, 7]. As observed in other cases, methylation level is variable along the repetitive sequence and hypomethylation is clustered at the 5’ end (DR1, <35 % of methylated CpGs and 5’, < 55 % of methylated CpGs) of the repeat in the affected members of the family (I1 and II1). Interestingly, hypomethylation is more pronounced in muscle and fibroblasts than in blood. Altogether, these data suggest that in this family, FSHD is genetically associated with *SMCHD1* mutation and D4Z4 hypomethylation, rather than D4Z4 copy number.

Both wild-type and mutated transcripts were detected by RT-PCR. The 7 bp insertion is predicted to activate an exonic cryptic acceptor site, however such altered splicing was not evident in cells available from the patient. At the protein level, the insertion would cause a frameshift with the presence of premature stop codon 4 amino acids after the insertion. In western blot, the truncated protein was undetectable and the quantity of SMCHD1 protein was comparable between the patient and controls thus making haploinsufficiency improbable. The *SMCHD1* mutation has been transmitted from the mother to one daughter and segregates with the presence of the symptoms. No transmission has been observed in the second daughter and brother who are not affected. Interestingly, we analyzed if other muscular gene variants might contribute to the FSHD phenotype in this family. Rare or unreported variants with a genetic status in proband and mother consistent with the age of disease appearance and/or severity are detailed in Additional file 2: Table S1. In particular, we describe a never reported heterozygous missense mutation in the *TTN* gene (c.8168 A > C; p.D2723A) which might act as a phenotype modifier.

In 80 % of the FSHD2 patients, the pathology has been associated with mutation in the *SMCHD1* gene. Most of the variations described so far are single base deletions, insertions, missense or nonsense mutations and splice site variants [8–13]. Larger insertions such as the one described here are rare and a single case of insertion of 7 nucleotides in exon 42 has been reported so far (LOVD SMCHD1 variant database). Interestingly, the different variations are mainly clustered in exons 9-12 and exons 25-37, proximal to the two functional domains, the “Histidine Kinase-like ATPase domain” encompassing exons 2-8 and the “Flexible Hinge domain” from exon 41 to exon 45, but not within these domains [8, 9, 11]. Only 4 mutations have been identified within these functional domains, three in the ATPase domain and one in the flexible Hinge domain [8, 9, 11]. The variation reported here corresponds to a duplication of a short TATAATA sequence within exon 37, proximal to the hinge domain. This insertion induces a frameshift and the appearance of stop codon 4 amino acids after the insertion. We demonstrate that this insertion leads to the production of a mutated transcript detectable in muscle, fibroblasts and PBMCs in patients but does not correlate with a detectable modification in the SMCHD1 protein level when probed with antibodies directed either against the ATPase or Hinge domains or the appearance of a shorter isoform (1543 amino acids long) as expected for a truncating mutation in both fibroblasts and PBMCs suggesting that the truncated protein might be degraded. However, treating patient’s fibroblasts with an NMD inhibitor did not show specific rescue of the mutated transcript.

## Conclusion

In this study, we demonstrate that insertion of a TATAATA DNA motif within the exon 37 of SMCHD1 leads to the production of a mutant transcript detectable in the different tissues analyzed that is not specifically degraded by NMD. However, at the protein level, we did not observe a clear decrease in the protein level, which was comparable between affected and control individuals. *SMCHD1* mutation, together with methylation level at D4Z4 segregates in the affected members of the family thus representing the strongest genetic candidates for FSHD in this family. Nevertheless, for the proband, the DUX4-fl transcript, which is only amplified in approximately 50 % of the muscle biopsies tested [26, 27] is not present at a detectable level in muscle biopsy. Surprisingly, however, DUX4-fl transcript is present in primary fibroblasts.

In this family, *SMCHD1* haploinsufficiency is associated with D4Z4 hypomethylation. However, how this haploinsufficiency triggers disease onset remains to be established. Although speculative, we cannot exclude a phenotype modifier effect by other muscle gene variants, like the *TTN* heterozygous p.D2723A variant. In addition, besides its role in D4Z4 methylation, SMCHD1 might contribute to the *4q35* higher-order chromatin conformation and long-range regulation of other *cis*-acting genes such as *FRG1* [28], *FAT1* [29, 30] or *SORBS2* [31] or activate DUX4-Fl production and subsequent target activation [32, 33] at early stages [26, 32].

## Additional files

Additional file 1: Figure S1. Schematic representation of the bar code used for molecular combing analyses. A. The 4qA and 4qB haplotypes correspond to different genomic elements. Chromosome 4A and 10 share the distal region as well as a 42 kb region upstream of the D4Z4 repeat array, including the p13E-11 sequence used as a probe for Southern blot. Further upstream sequences, starting with the inverted D4Z4 repeat array, are specific to either 4q or 10q. The bar-code used to distinguish the three different alleles is based on a combination of three different colors and different DNA probes encompassing the distal regions up to the telomeric sequence [19]. The 3-color barcode was previously described and comprises 2 probes detected in blue, which hybridize the proximal region common to chromosomes 4 and 10, one 6 kb probe detected in red, which hybridizes in the (TTAGGG)n telomeric extremities, and a probe labeled in red that hybridizes the qA-specific β-satellite region, with a variable length (1–5 kb). The qB-specific probe, immediately adjacent to D4Z4, is detected in blue. (TIFF 1521 kb) Additional file 2: Table S1. Genetic variants in gene associated with neuromuscular disorders in the proband (P) and her mother (M). **Table S2.** *In silico* prediction of the different genetic variants and mode of inheritance in neuromuscular disorders (AD, autosomal dominant ; AR, autosomal récessive ; XLR, X-linked Recessive). (DOCX 110 kb) Additional file 3: Figure S2.
**A.** Schematic representation of the *SMCHD1* gene. **B.** Analysis of the SMCHD1 mutation segregation among family members. I1 and II1 carry the same heterozygous insertion (c.4614_4615 insTATAATA). **C.** Analysis of the heterozygous duplication of 7 nucleotides in exon 37 of *SMCHD1* at the mRNA level after cloning and sequencing of the two *SMCHD1* alleles from II1. **D.** Predictive analysis for a putative ORF within the r.4614_4615 insTATAATA cDNA. Top: wild type ORF; bottom: predicted ORF terminating by a premature termination codon (p.A1539Yfs*4) in exon 37. Nucleotide positions are given using the *SMCHD1* NM_015295 reference sequence. (TIFF 1521 kb)

## Abbreviations

FCS: Fetal calf serum
FSHD1: Facio Scapulo Humeral Dystrophy Type 1
FSHD2: Facio Scapulo Humeral Dystrophy Type 2
NMD: Non-sense mediated mRNA decay
PBMC: Peripheral blood mononuclear cells
PBS: Phosphate buffered solution
PRC: Polycomb repressive complex
RT-PCR: Reverse transcription polymerase chain reaction
RU: Repeated units
SDS: Sodium dodecyl sulfate
SMC: Structural maintenance of chromosomes
SMCHD1: Structural maintenance of chromosomes flexible hinge domain containing 1
TrimethylH3K27: Trimethylated histone 3 lysine 27
