# Supplementary figures and images for: Segregation between SMCHD1 mutation, D4Z4 hypomethylation and Facio-Scapulo-Humeral Dystrophy: a case report
Source: BMC Med Genet. 2016 Sep 15;17:66. doi: 10.1186/s12881-016-0328-9 (PMC5025538; doi:10.1186/s12881-016-0328-9)

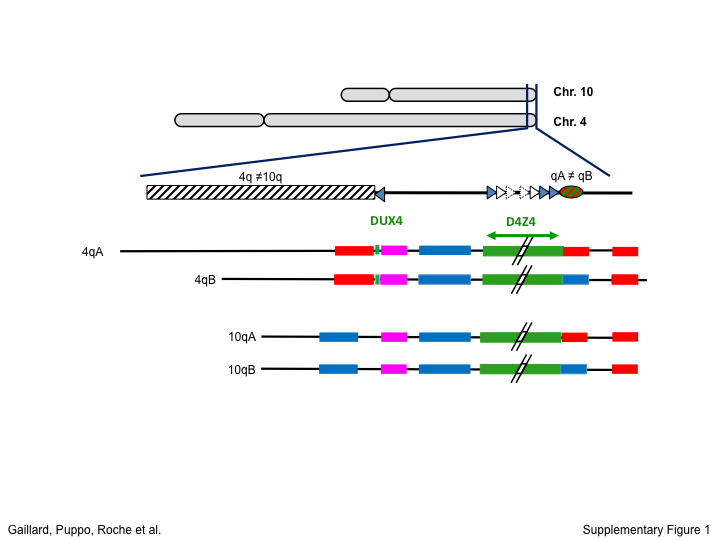

Supplement: Additional file 1: Figure S1. — Schematic representation of the bar code used for molecular combing analyses. A. The 4qA and 4qB haplotypes correspond to different genomic elements. Chromosome 4A and 10 share the distal region as well as a 42 kb region upstream of the D4Z4 repeat array, including the p13E-11 sequence used as a probe for Southern blot. Further upstream sequences, starting with the inverted D4Z4 repeat array, are specific to either 4q or 10q. The bar-code used to distinguish the three different alleles is based on a combination of three different colors and different DNA probes encompassing the distal regions up to the telomeric sequence [19]. The 3-color barcode was previously described and comprises 2 probes detected in blue, which hybridize the proximal region common to chromosomes 4 and 10, one 6 kb probe detected in red, which hybridizes in the (TTAGGG)n telomeric extremities, and a probe labeled in red that hybridizes the qA-specific β-satellite region, with a variable length (1–5 kb). The qB-specific probe, immediately adjacent to D4Z4, is detected in blue. (TIFF 1521 kb) [file 12881_2016_328_MOESM1_ESM.tiff]

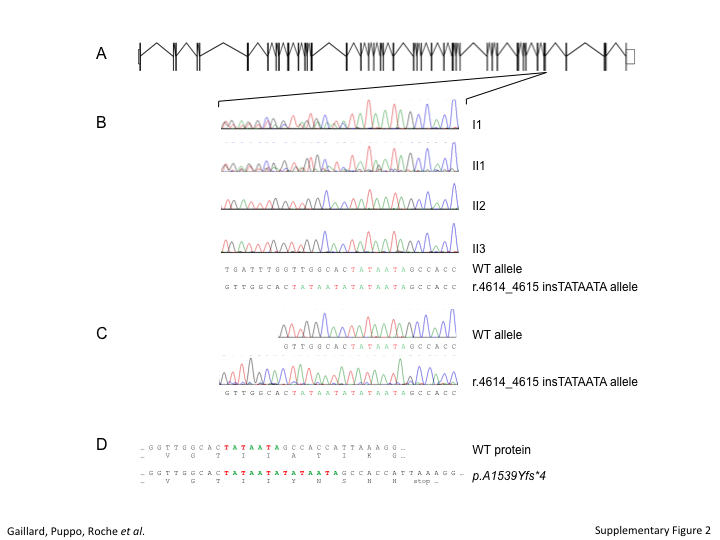

Supplement: Additional file 3: Figure S2. — A. Schematic representation of the SMCHD1 gene. B. Analysis of the SMCHD1 mutation segregation among family members. I1 and II1 carry the same heterozygous insertion (c.4614_4615 insTATAATA). C. Analysis of the heterozygous duplication of 7 nucleotides in exon 37 of SMCHD1 at the mRNA level after cloning and sequencing of the two SMCHD1 alleles from II1. D. Predictive analysis for a putative ORF within the r.4614_4615 insTATAATA cDNA. Top: wild type ORF; bottom: predicted ORF terminating by a premature termination codon (p.A1539Yfs*4) in exon 37. Nucleotide positions are given using the SMCHD1 NM_015295 reference sequence. (TIFF 1521 kb) [file 12881_2016_328_MOESM3_ESM.tiff]
