# Supplementary material for: Segregation between SMCHD1 mutation, D4Z4 hypomethylation and Facio-Scapulo-Humeral Dystrophy: a case report
Source: BMC Med Genet. 2016 Sep 15;17:66. doi: 10.1186/s12881-016-0328-9 (PMC5025538; doi:10.1186/s12881-016-0328-9)
Supplement: Additional file 2: Table S1. — Genetic variants in gene associated with neuromuscular disorders in the proband (P) and her mother (M). Table S2. In silico prediction of the different genetic variants and mode of inheritance in neuromuscular disorders (AD, autosomal dominant ; AR, autosomal récessive ; XLR, X-linked Recessive). (DOCX 110 kb) [file 12881_2016_328_MOESM2_ESM.docx]

**Supplementary table 1.** Genetic variants in gene associated with neuromuscular disorders in the proband (P) and her mother (M).

| Chr | GRCh38.2 position | Gene | Refseq | ORF variation | Protein variation | Sample | Status | Genotype | rs | ExAc minor allele freq | 1000G variant Freq (%) |
| --- | --- | --- | --- | --- | --- | --- | --- | --- | --- | --- | --- |
| Chr2 | g.178770624 | *TTN* | NM_001267550 | c.8168A>C  (exon35, 264bp) | D2723A | M/P | hom_ref het | TT GT | N/A |  |  |
| Chr14 | g.64074004 | *SYNE2* | NM_182914 | c.10734G>T  (exon53, 169bp) | V3578V | M/P | hom_ref het | GG GT | rs756765132 | 0.00002 | N/A |
| Chr14 | g.64098818 | *SYNE2* | NM_182914 | c.12378C>T  (exon63, 75bp) | S4126S | M/P | hom_ref het | CC CT | rs36007735 | 0.006 | 0.0024 |
| Chr2 | g.151525997 | *NEB* | NM_001164507 | c.22122C>G  (exon150, 111bp) | D7374E | M/P | hom_ref het | GG CG | rs192402741 | 0.003 | 0.0008 |
| Chr2 | g.151568420 | *NEB* | NM_001164507 | c.17635-3T>G  (exon112, 102bp) | N/A | M/P | hom_diff het | -/-  -/A | rs111853129 | N/A | N/A |
| Chr2 | g.151727802-151727816 | *NEB* | NM_001164507 | c.169_183delCTGGCACAGCCAGCA | p.157_A61del | M/P | het het | TGCTGGCTGTGCCAG/- TGCTGGCTGTGCCAG/- | rs377452683, benign in ClinVar | 0.006 | 0.02 |
| ChrX | g.32491508 | *DMD* | NM_004006 | c.2391T>G  (exon20, 242bp) | N797K | M/P | het het | AC AC | rs72468681 | 0.008 | 0.003 |
| Chr21 | g.46126176 | *COLA6A2* | NM_001849 | c.2361G>A  (exon26, 453bp) | T787T | M/P | hom_ref het | GG AG | rs566966690 | N/A | N/A |

**Supplementary table 2.** *In silico* prediction of the different genetic variants and mode of inheritance in neuromuscular disorders (AD, autosomal dominant ; AR, autosomal récessive ; XLR, X-linked Recessive).

| **Chr** | **Gene** | **ORF variation** | **Splicing predictions** | **UMD predictions** | **Muscle disease inheritance** | **Reported variants** |
| --- | --- | --- | --- | --- | --- | --- |
| Chr2 | *TTN* | c.8168A>C  (exon35, 264bp) | Predicted New ESS and ESE Broken. If skipped exon in frame. | Pathogenic | LGMD2J (AR) | N/A |
| Chr14 | *SYNE2* | c.10734G>T  (exon53, 169bp) | Predicted ESE Broken. | Polymorphism | AD | N/A |
| Chr14 | *SYNE2* | c.12378C>T  (exon63, 75bp) | Predicted New Donor and ESS | Polymorphism | AD | Clin Var: Benign  LOVD: Reported by Lehtokari et al, 2014 [[1](#_ENREF_1)]. |
| Chr2 | *NEB* | c.22122C>G  (exon150, 111bp) | Predicted New Acceptor and ESS. ESE Broken | Polymorphism | AR | N/A |
| Chr2 | *NEB* | c.17635-3T>G  (exon112, 102bp) | Altered Acceptor Site | N/A | AR | N/A |
| Chr2 | *NEB* | c.169_183delCTGGCACAGCCAGCA | N/A | N/A | AR | N/A |
| ChrX | *DMD* | c.2391T>G  (exon20, 242bp) | Predicted New Acceptor and ESE Broken | Pathogenic | XLR | N/A |
| Chr21 | *COLA6A2* | c.2361G>A  (exon26, 453bp) | Predicted ESE Broken If skipped exon in frame. | Polymorphism | AR, AD | Clin Var: Benign |

1. Lehtokari VL, Kiiski K, Sandaradura SA, Laporte J, Repo P, Frey JA et al. Mutation update: the spectra of nebulin variants and associated myopathies. Hum Mutat. 2014;35(12):1418-26. doi:10.1002/humu.22693.
